# Visual appearance of the virtual hand affects embodiment in the virtual hand illusion

**DOI:** 10.1038/s41598-020-62394-0

**Published:** 2020-03-25

**Authors:** Maria Pyasik, Gaetano Tieri, Lorenzo Pia

**Affiliations:** 1 Spatial, Motor and Bodily Awareness research group, Department of Psychology, University of Turin, Turin, Italy; 2 Virtual Reality Lab, Unitelma Sapienza of Rome, Rome, Italy; 3 IRCSS Santa Lucia Foundation, Rome, Italy; 4 Neuroscience Institute of Turin, Turin, Italy

**Keywords:** Consciousness, Perception

## Abstract

Body ownership (the feeling that one’s body belongs to oneself) is commonly studied with Rubber hand illusion (RHI) paradigm that allows inducing a temporary illusory feeling of ownership of a life-sized rubber hand. However, it remains unclear whether illusory ownership of the fake hand relies on the same mechanisms as ownership of one’s own real hand. Here, we directly compared ownership of the own hand (OH) and fake hand (FH) in the same set of conditions within immersive virtual reality. We obtained behavioral (proprioceptive drift) and subjective (questionnaire) measures of ownership and disownership for virtual OH, FH and object (Obj) that were located congruently or incongruently with the participant’s real hand and were stimulated synchronously or asynchronously with the real hand. Both OH and FH (but not Obj) were embodied after synchronous stimulation in both locations. Crucially, subjective ownership of the OH was stronger than of the FH in congruent location after synchronous stimulation. It was also present after asynchronous stimulation, being stronger when the virtual OH was subjectively more similar to the real hand. The results suggest that the detailed appearance of the body might act as an additional component in the construction of body ownership.

## Introduction

The experience that one’s own body belongs to oneself, i.e. body ownership^1^, is one of the crucial components of human self-awareness, and it is constant, omnipresent and largely unconscious^2^.

Body ownership can be investigated either in neurologically-based disorders of body representation, e.g.,^3–8^, or in healthy participants by using a paradigm called Rubber hand illusion (RHI^9^). During the RHI procedure, a life-sized rubber hand is placed in front of the participant congruently with the participant’s body (i.e., in first-person perspective), while the corresponding real hand is hidden from the view. The rubber and the real hands are touched synchronously in space and time (e.g., stroked by paintbrushes), which typically evokes a vivid illusory sensation that the fake hand became a part of participant’s own body. Furthermore, embodiment of the rubber hand is additionally characterized by the feeling of disownership/disembodiment of the corresponding own hand, i.e., the feeling that one’s own hand disappeared when the rubber hand was embodied^10,11^. The illusory experience is quantified at subjective (questionnaire answers), behavioral (proprioceptive drift, i.e. the perceived mislocalization of one’s own hand towards the fake hand) and physiological level (e.g., skin conductance response^12,13^ and hand temperature^14^). Overall, stronger embodiment illusion over the fake hand is therefore characterized by higher ownership and disownership ratings, larger proprioceptive drift and decrease in the own-hand temperature.

The RHI effects are explained by the fact that when the incoming visual, tactile and proprioceptive signals are temporally congruent (synchronous stimulation), the conflict between vision of the fake hand and tactile and proprioceptive sensation from one’s own hand is resolved in favor of vision^15–17^. The explanation is consistent with a well-known neurocognitive model of body ownership during the RHI^17^. The model states that body ownership is constructed at a series of hierarchical stages where incoming sensory signals that constantly reach the body are compared with preexisting internal body representations. At the end, a full and coherent ownership of a fake hand is constructed. Importantly, when the fake hand is embodied, its representation could partially replace the representation of the real hand, instead of simply becoming an additional, third, limb (e.g.,^14^; although some studies demonstrated illusory ownership of supernumerary limbs, e.g.,^18^).

Since the first time it was described, the RHI paradigm has been adapted for different technologies, such as robotic devices or virtual reality, resulting in Robot hand illusion^19–22^, Virtual hand-^23–26^ and Virtual body illusion^27,28^. Importantly, differently from the classical RHI setup where the fake hand is usually placed near the real hand, in a virtual reality setup, the virtual hand can be placed in the same position as the real one, giving the experimental advantage of the congruence in visuo-proprioceptive information. Indeed, recent studies demonstrated that, in a VR setup, the simple passive observation of a virtual limb (without visuo-tactile stimulation) is a sufficient condition for eliciting illusory ownership over the virtual limb itself^24,25,28–31^. Furthermore, such enhanced role of visual information from the virtual scene can provide ample opportunities for experimental manipulations of body ownership by maintaining the visuo-proprioceptive information constant. For example, it allows creating a mismatch between the movements performed by the virtual and the real body, which the participant remains unaware of^32,33^.

The multitude of RHI studies draw conclusions about body ownership from an artificial experimental situation of illusory ownership of a fake/virtual hand/body. In contrast, some studies manipulated participants’ own hand in RHI-like setups. For instance, Projected hand illusion^34,35^ uses the videos of participant’s own hand that are projected on a table in front of them, either in live timing, or with a delay (which creates asynchronous stimulation). In more immersive setups, such as the MIRAGE system, the videos of the own hand are projected on the same plane as the real hand^18^, or presented in a head-mounted display (HMD)^36,37^. Moreover, other variations of the experimental procedure used augmented-^38^ and mixed reality^10^. Such manipulations of the own hand allow not only evaluating the sense of ownership, but also the sense of disownership of one’s own hand across various conditions of multisensory conflict, which usually involve the dissociation between vision and touch in terms of timing or location, as well as between the seen and perceived hand position. Indeed, when incongruent (e.g., temporally or spatially asynchronous) stimulation was applied to the participant’s own hand, they experienced disownership of their hand that was represented by subjective, physiological and neural measures^10,36^. As for the sense of ownership, the conclusions of these studies were largely similar to the studies with the fake hand/body, such as the importance of multisensory integration in the construction of body ownership.

So far, however, no study has compared ownership/disownership of one’s own vs other hand in an identical complete set of conditions that would include different locations of the seen hand with respect to the real hand, and different types of stimulation. Therefore, a clear comprehension of the role played by visual information in eliciting ownership over a fake hand is still lacking.

To fill in this gap, here, we integrated 3d-scans of participant’s own hand within a setup in immersive virtual reality (IVR) and manipulated the appearance of the virtual hand [own hand (OH), fake hand (FH) and a hand-sized object resembling a plain wooden block (Obj^25^) as a control condition], its location [congruent (Congr) or incongruent (Incongr) with the participant’s real hand] and type of visuotactile stimulation [synchronous (Syn) or asynchronous (Asyn)]. We then performed a RHI-like procedure in each condition and registered behavioral (proprioceptive drift) and subjective (questionnaire) measures of ownership of the virtual hand and disownership of the own hand.

We hypothesized that the illusory effects would be the strongest in the conditions with the highest congruence of multisensory information (vision, touch, proprioception, hand position and appearance) and would decrease with every degree of incongruence. By systematically varying the three factors described above (hand appearance, visuotactile stimulation and hand location), we therefore expected to identify, which of the factors played a more important part in constructing the feeling of ownership. We predicted that, firstly, subjective ownership of the virtual FH would be present only in Syn stimulation conditions (both in Congr and Incongr location), as in the typical RHI. Secondly, compared to the FH, ownership of the OH would be stronger after Syn stimulation, especially in the Congr location, but it also might be present, to some extent, in the conditions of Asyn stimulation in both locations. Thirdly, the proprioceptive drift would be present only in the conditions with Incongr location of OH or FH after Syn stimulation. As for the subjective disownership of the real hand, we expected to find some degree of disownership of the own hand in the FH conditions after Syn, but not Asyn stimulation, both in Congr and Incongr location. In turn, for the OH, disownership might be stronger in the conditions of Asyn stimulation and Incongr location. Finally, none of the conditions that include virtual Obj should induce neither subjective ownership/disownership, nor the proprioceptive drift.

## Methods

### Participants

Forty-five right-handed^39^ healthy participants (32 females, 13 males, mean age ± SD = 22.96 ± 2.48) with normal or corrected-to-normal visual acuity and no history of neurological or psychiatric disease took part in the study. All participants were naïve to the experimental procedure and to the purpose of the study and provided written informed consent to participate in the study. The study was approved by the Bioethical Committee of the University of Turin and was carried out in accordance with the ethical standards of the 2013 Declaration of Helsinki.

### Experimental design

**Figure 1:**
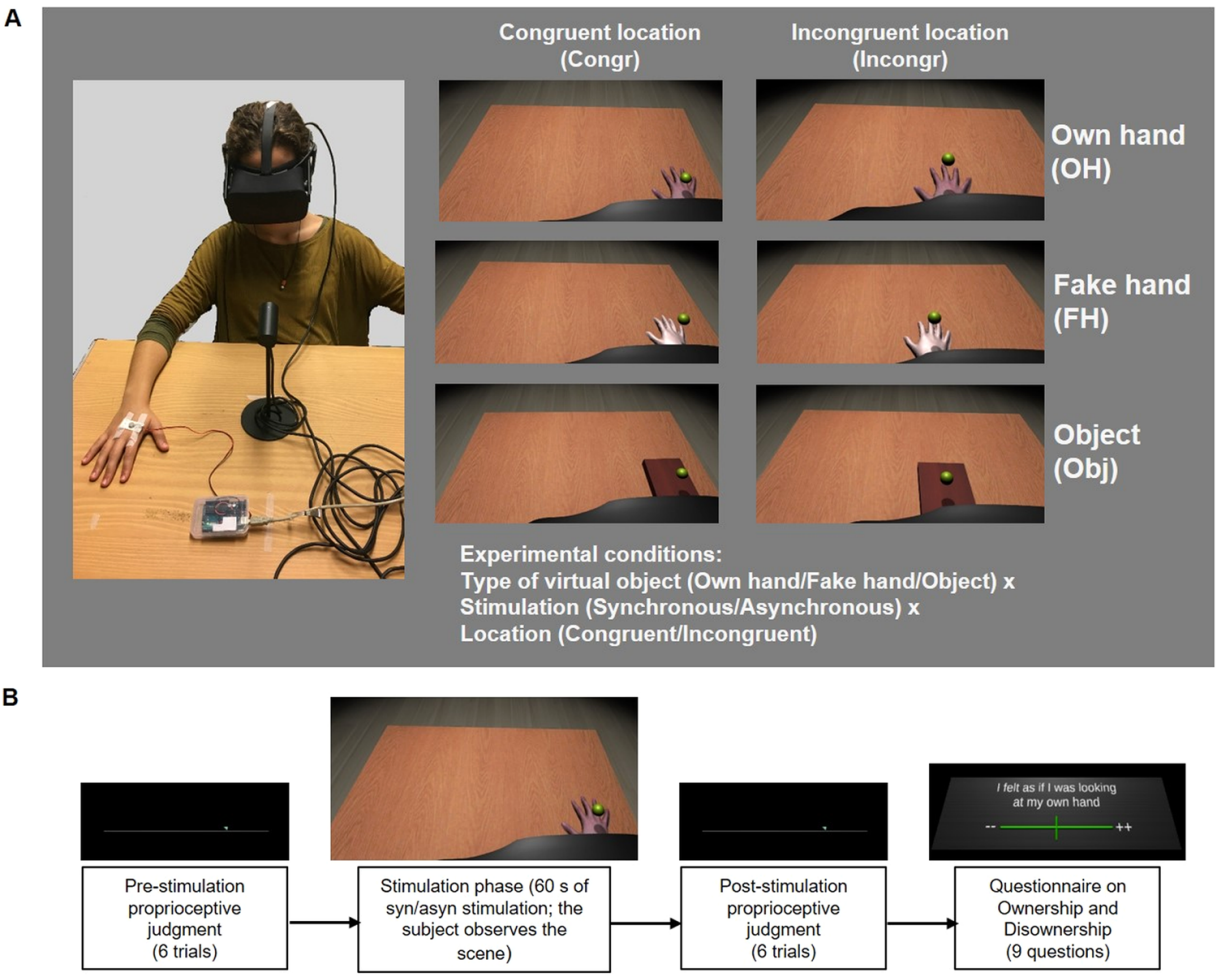
Experimental setup and procedure: (**A**) setup; the left panel shows the participant wearing the HMD, right panel shows the participant’s view of the virtual scene in different conditions – Own hand (top row), Fake hand (middle row), Object (bottom row), and the list of experimental conditions; (**B**) timeline of a single condition.

The experiment included the VHI procedure that was modified across twelve experimental conditions in a within-subject design: 3 × 2 × 2, i.e., Type of virtual object [own hand (OH), fake hand (FH), object (Obj)] x Location [congruent (Congr) with the participant’s real hand or incongruent (Incongr), shifted 20 cm towards the body midline] x Visuo-Tactile Stimulation [synchronous (Syn) or asynchronous (Asyn)] (see Fig. 1A, right panel). The experiment was conducted in a single session that lasted approximately two hours.

#### Stimuli

Before the experiment, we obtained 3D-scans of each participant’s right hand using a 3D scanner (Sense 3D Scanner, 3D Systems, Inc.); during the 3D-scanning, the participant’s hand was placed on a tabletop in a relaxed position. The scans were preprocessed with Sense software (3D Systems, Inc.) and 3DS Max 2015 (Autodesk, Inc.) and implemented in the virtual setup (see details below). The individual 3D-scan of participant’s hand was used for the OH conditions, while for the FH conditions, a gender-matched 3D scan of another person’s hand was used. In order to make the FH look more artificial, we increased the smoothness of the 3D scan by 50% and changed the color making it whiter than a human hand (RGB: 255; 212; 208). As a control condition, a virtual object (Obj), resembling a plain wooden block with the same dimensions as FH and OH, was designed in 3DS Max 2015 (Autodesk, Inc.) and implemented in the virtual scenario (see Fig. 1A, right panel). In accordance with previous evidence that the RHI susceptibility was not related to the hand laterality^40–42^, during the experiment we tested only the dominant (right) hand.

#### Setup

The virtual scenario was designed using 3DS Max 2015 (Autodesk, Inc.) and implemented in Unity 5 game software environment (http://unity.com). It was presented by means of Oculus Rift head-mounted display (HMD, www.oculus.com) with a 110° field-of-view (diagonal FOV) and a resolution of 2160 × 1200. The virtual scenario included a dark room with a table (90 × 60 cm, scale 1:1) having the same dimensions and color as the real table in the laboratory where the experiment took place. A virtual hand/object was positioned on the table and a virtual black cloth covered the distance between the hand’s wrist/the near edge of the object and the approximate location of the participant’s neck (as in the RHI, e.g.^9^). In order to deliver the vibrotactile stimulation on participant’s hand, a small round stimulator was attached to the back of participant’s right palm; during the experimental conditions, it was triggered by an Arduino microcontroller (https://www.arduino.cc) by means of a customized script implemented in Unity.

#### Procedure

Experimental setup and procedure are presented in Fig. 1. Written informed consent has been given by the person shown in the images to publish these in this online open-access publication.

After the virtual scene was adjusted for the participant, i.e., the 3D-scan of his/her hand was integrated in the scene, the participant was asked to sit in front of the table and put on the HMD. Participant’s right hand was placed on a fixed spot on the table (30 cm to the right from the middle of the table and the participant’s body midline). Then, participant was asked to sit comfortably and keep the hand relaxed and still during all experimental conditions.

Before the beginning of the experimental phase, the participants observed the virtual scene with the 3D-scan of their own hand located on the virtual table in the same spot where their real hand was. Since the experiment aimed at comparing ownership of the OH vs FH, it was crucial that the participants recognized their hand in the 3D image. Thus, they were asked to evaluate the similarity of the 3D image to their real hand, along with the plausibility of the virtual-hand’s location, and to rate the level of similarity on a 0–7 Likert scale (0 = absence of similarity and 7 = full correspondence to the appearance their real hand). Before the beginning of the data collection, we established that participants who rated the similarity of the virtual hand as less than 5/7 would not continue with the experimental tasks. None of the participants were excluded (the ratings of the virtual OH similarity ranged from 5 to 7, mean ± SD = 5.81 ± 0.68).

All experimental conditions had an identical experimental procedure that included: (i) pre-stimulation proprioceptive judgments, (ii) stimulation phase, (iii) post-stimulation proprioceptive judgments and (iv) questionnaire about the sense of ownership and disownership (see Fig. 1B). In turn, the conditions differed in three factors: (1) Type of virtual object: OH, FH and Obj; (2) Location: each of these objects was located either congruently with the participant’s real hand (i.e., the object’s position on the virtual table fully corresponded with the location of the real hand), or incongruently (i.e., the object was shifted 20 cm to the left, towards the middle of the virtual and real table and towards the participant’s body midline); (3) Visuotactile stimulation: for each type of object in each location, there were two types of visuotactile stimulation – Syn and Asyn. During the stimulation phase, a virtual ball appeared in the virtual scenario and moved up and down (according to a preprogrammed animation), touching the virtual object in the location that corresponded with the location of the vibrotactile stimulator on the participant’s hand (see Fig. 1A) (frequency and duration of each touch were 0.27 Hz and 500 ms, respectively). In Syn stimulation conditions, the stimulator on the participant’s hand vibrated at the moment when the virtual ball touched the object. In Asyn stimulation conditions, the same vibrotactile stimulation was delivered in the phase opposite to the ball’s touch, i.e., when the ball was up in the air.

In each condition, firstly, the virtual scene was obscured and the participants saw only a white horizontal line with a green cursor on a black background. The line was 100 cm long, therefore slightly exceeding the length of the real and the virtual table. The participants were asked to move the cursor along that line (using left and right arrow buttons with their left hand) so that it would point to the perceived position of their real right middle finger. This procedure was repeated for six trials; in the beginning of every trial, the cursor appeared at a random position on the line. The actual location of the participant’s middle finger was then subtracted from the subjective responses (i.e., the registered location of the cursor) and the resulting differences were referred to as *pre-stimulation proprioceptive judgments*.

Then, the white horizontal line and the cursor were replaced by the virtual scene. The participants were instructed to pay attention the scene and remain still. The scene included the *stimulation phase* (60 s). After the stimulation phase, the virtual scene was obscured again and the white line with the cursor appeared. As in the pre-stimulation proprioceptive judgments, the participants had to move the cursor to indicate the perceived location of their right middle finger (6 trials). The differences between actual and perceived location of the finger were referred to as *post-stimulation proprioceptive judgments*.

Post-stimulation proprioceptive judgments were followed by the ownership and disownership questionnaire. Each statement of the questionnaire was presented separately on the black background; below the statement, the participants saw a visual analogue scale (VAS) with the cursor placed in the middle and the ends marked with “−” (left side) and “+” (right side). The participants were told that the statements refer to the last scene that they saw (in order to avoid confusion between the conditions). They were then asked to read each statement carefully and express the level of agreement or disagreement by moving the cursor along the VAS; the middle of the scale represented uncertainty, the left end – complete disagreement and the right end – complete agreement. As soon as the participants responded to the presented statement, it was replaced with the next one. The recorded responses represented the cursor position on the VAS, with the scale ranging from −50 to 50. The questionnaire contained nine statements – three Ownership statements (describing different aspects of the feeling of ownership of the virtual hand/object), three disownership statements (describing different aspects of the feeling of disownership of one’s own hand) and three Control statements (included for controlling participant’s compliance with the instructions and suggestibility effects). The statements were selected from previous studies^25,36,43^ and modified in order to be suitable for all conditions (see Table 1). The order of the statements was randomized in each condition.

**Table 1:** Ownership and disownership questionnaire.

**Ownership**	Q1. I felt as if I was looking at my own hand Q2. I felt as if the Virtual Hand/Virtual Object was part of my body Q3. It felt as if the touch I experienced was directly caused by the ball that was touching the virtual hand/object
**Control**	Q4. It felt as if I had more than one right hand Q5. I felt as if my real hand was turning virtual Q6. It felt as if the touch I experienced came from somewhere between my own hand and the virtual hand/object
**Disownership**	Q7. It seemed as if my hand had disappeared Q8. It seemed as if I could not really tell where my hand was Q9. It seemed as if I was unable to move my hand

The order of conditions was counterbalanced across participants according to a Latin Square. Between the conditions, participants were asked to lift their right hand from the table and move it in order to avoid possible aftereffects of the previous conditions. Prior to beginning each condition, the experimenter placed the participant’s hand back on the marked spot on the table and asked the participant to keep the hand relaxed and still throughout the following task.

### Data handling

#### Questionnaire

We calculated the general Ownership, Control and Disownership scores by averaging the scores in (Q1–Q3), (Q4–Q6) and (Q7–Q9), respectively. We then compared Ownership and Control scores within each condition, and Ownership scores between Syn and Asyn stimulation within each condition. Furthermore, we compared the ownership scores between different types of virtual object and location with Syn stimulation conditions. We did not perform an identical analysis for Asyn stimulation, since the preliminary analysis showed that the ownership ratings were negative in all Asyn conditions.

Then, in order to obtain a more detailed pattern of differences between conditions, we analyzed separate ownership statements (Q1–Q3), since they represent different aspects of ownership^11^. We followed the same logic, i.e., firstly, we compared the scores between Syn and Asyn stimulation within each condition, then between conditions with Syn stimulation (as for the averaged ownership ratings, the Asyn stimulation conditions were not analyzed in detail due to the negative ratings in the majority of conditions).

We followed a similar logic in analyzing Disownership statements, i.e., performed the initial comparisons between Syn and Asyn stimulation in each condition and then compared the ratings between different types of virtual object and location with Syn stimulation conditions.

As for Control statements, since the preliminary analysis showed that the scores in all conditions were negative, we did not perform any detailed analyses.

#### Proprioceptive drift

In each condition, pre-proprioceptive judgments were subtracted post-stimulation proprioceptive judgments, and the resulting differences were averaged between the six trials. This value was referred to as proprioceptive drift^35^. It was measured in cm; positive values represented the perceived shift of the participant’s hand to the left, i.e., towards the body midline and towards the virtual object in the conditions with the incongruent location. Negative values, therefore, represented the perceived shift of the hand away from the body midline. The proprioceptive drift was firstly compared between Syn and Asyn condition separately for each type of virtual object and each location. Then, we compared the drift between all conditions with Syn stimulation and between all conditions with Asyn stimulation, separately for Congr and Incongr location.

#### Correlations

We calculated correlations between the subjective ratings of OH similarity (reported prior to the experiment, as described above) and the proprioceptive drift and ownership ratings.

Since at least one variable in each analysis violated the criteria for normality of distribution on a Shapiro–Wilk test (p > 0.05), nonparametric analyses were performed. The effect sizes were estimated with correlation coefficient effect size *r*. Bonferroni correction for multiple comparisons was performed for adjusting the alpha level for statistical significance according to the number of comparisons in each analysis.

## Results

Due to the large number of performed analyses and generated results, below we present their summary. Detailed results and descriptive statistics can be found in Supplementary Materials.

### Subjective reports on ownership

**Figure 2:**
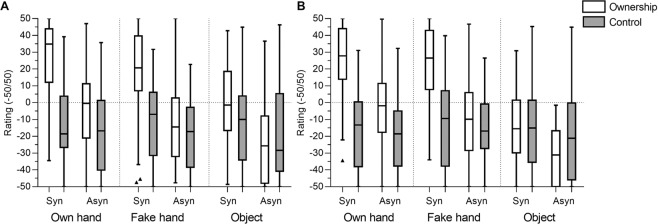
Questionnaire scores (ownership and control; −50/+50): (**A**) Congr Location; (**B**) Incongr Location. The box represents the first and the third quartile with the line in the middle of the box representing the median, and the bottom and top whiskers represent the first quartile minus 1.5*IQR and the third quartile plus 1.5*IQR.

Averaged Ownership and Control ratings are presented in Fig. 2. The comparisons between Ownership and Control scores within each condition showed significantly higher Ownership ratings in all OH conditions (both locations and types of stimulation) and FH with Syn stimulation (also in both locations), but not for FH with Asyn stimulation or Obj. As for the comparison of ownership ratings between Syn and Asyn stimulation conditions for each type of object, they were significantly higher after Syn stimulation compared to Asyn stimulation in every condition. Within the Syn stimulation conditions, comparable ownership was observed for OH and FH both in Congr and Incongr location (Congr: p = 0.016 [N.S. after Bonferroni correction; alpha level: p = 0.006]; Incongr: p = 0.56). In turn, both OH and FH had significantly higher ownership ratings then Obj in both locations (Congr location, OH: p < 0.0001, r = 0.48, FH: p < 0.0001, r = 0.38; Incongr location, OH: p < 0.0001, r = 0.68; FH: p < 0.0001, r = 0.65). Neither OH, nor FH ratings differed between Congr and Incongr location, while for Obj, the ratings in Congr location were significantly higher compared to Incongr. Crucially, the ownership ratings were positive only for OH and FH with Syn stimulation, both Congr and Incongr location. Negative ratings in other conditions suggest the absence of subjective ownership.

**Figure 3:**
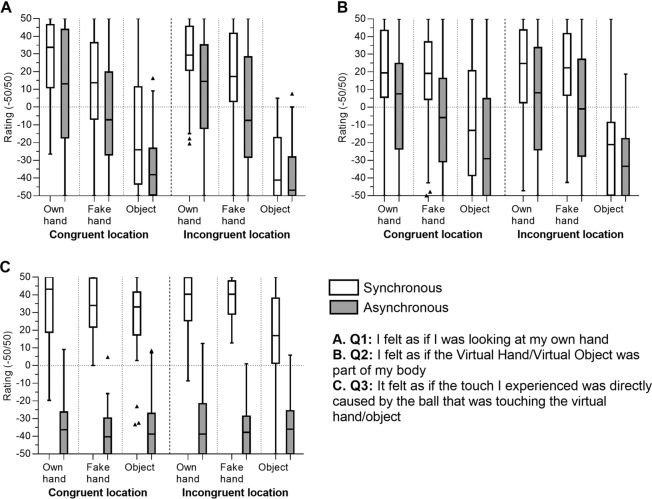
Ownership questionnaire scores (−50/+50): (**A**) Q1 (“*I felt as if I was looking at my own hand”*); (**B**) Q2 (*“I felt as if the Virtual Hand/Virtual Object was part of my body”*); (**C**) Q3 (“*It felt as if the touch I experienced was directly caused by the ball that was touching the virtual hand/object*). The box represents the first and the third quartile with the line in the middle of the box representing the median, and the bottom and top whiskers represent the first quartile minus 1.5*IQR and the third quartile plus 1.5*IQR.

In order to obtain more detailed results on ownership, we analyzed the three Ownership statements separately (see Fig. 3). In Q1 (*“I felt as if I was looking at my own hand”*), the ratings in Syn stimulation were significantly higher than in Asyn in all conditions, except for Obj Incongr. Further comparisons within the Syn stimulation conditions showed that the ratings were the highest for OH Congr compared to FH Congr and Obj Congr (OH vs FH: p < 0.0001, r = 0.58; OH vs Obj: p < 0.0001, r = 0.16), while for Incongr location OH and FH were comparable (p = 0.03 [N.S. after Bonferroni correction; alpha level: p = 0.006]) and significantly higher than Obj (p < 0.0001, r = 0.04). Importantly, they were positive only in all OH conditions (regardless of location and stimulation type) and FH Syn (in both locations).

In Q2 (*“I felt as if the Virtual Hand/Virtual Object was part of my body”*), similarly to Q1, the ratings were significantly higher in Syn stimulation than in Asyn in all conditions, except for Obj Incongr. Within the Syn stimulation conditions, OH and FH did not differ significantly in both locations (Congr: p = 0.99; Incongr: p = 0.79), and their ratings were positive and significantly higher compared to the Obj, which had negative ratings; the ratings were also positive in both OH Asyn conditions.

In Q3 (“*It felt as if the touch I experienced was directly caused by the ball that was touching the virtual hand/object*”), a similar pattern was observed in all conditions: the ratings after Syn stimulation were significantly higher than after Asyn stimulation in all conditions.). In Syn Congr conditions, the ratings for the three types of virtual object were not different, and in Syn Incongr conditions, OH and FH did not differ between each other but had significantly higher ratings than Obj. Within each type of virtual object, Congr condition did not differ significantly from Incongr condition. The ratings were positive in all Syn conditions and negative in all Asyn conditions.

### Proprioceptive drift

**Figure 4:**
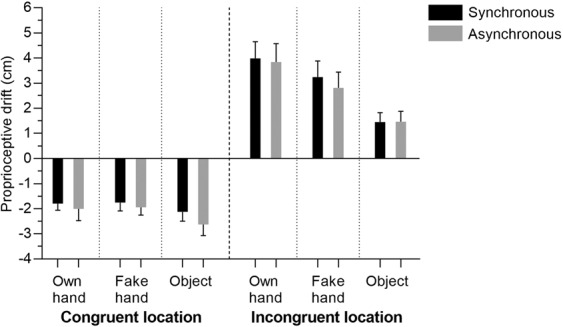
Proprioceptive drift (mean ± SEMs, cm).

According to the Wilcoxon signed rank test, proprioceptive drift was not significantly different between Syn and Asyn stimulation in any of the conditions; see Fig. 4. Furthermore, it was significantly higher for OH than for Obj in Incongr Syn condition (p = 0.001, *r* = 0.20), while no significant differences were present for Congr conditions.

It is also necessary to note that in all Congr conditions, the proprioceptive drift was negative, i.e., the perceived position of participant’s own hand shifted away from the body midline. On the contrary, in all Incongr conditions, it was positive, which suggests the perceived shift of participant’s hand towards the incongruently located virtual object and the body midline.

### Subjective reports on disownership

**Figure 5:**
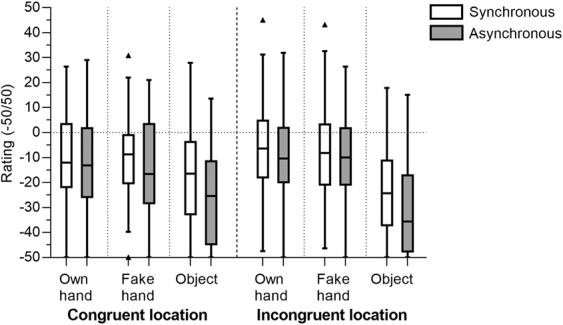
Disownership questionnaire scores (−50/+50). The box represents the first and the third quartile with the line in the middle of the box representing the median, and the bottom and top whiskers represent the first quartile minus 1.5*IQR and the third quartile plus 1.5*IQR.

The ratings in disownership questions (averaged Q7-Q9) were negative in all conditions (see Fig. 5), which suggests that the participants did not experience disownership of their own hand in any of the conditions. However, they were relatively higher for OH and FH conditions, especially in Incongr location, regardless of the stimulation type.

### Correlations

We calculated Spearman rank order correlations between the subjective ratings of virtual OH similarity to participant’s own real hand and the proprioceptive drift and ownership ratings, represented both by the averaged ownership ratings (Q1-Q3) and by the three statements separately. The only significant positive correlation was observed between the subjective OH similarity and the ratings in ownership Q2 in OH Congr Asyn condition (ρ = 0.45, p = 0.002). No significant correlations were present in other conditions either for the questionnaire, or for the drift. Therefore, stronger embodiment of the virtual OH after Asyn stimulation was observed in the participants that were more ready to recognize their own hand in its 3D scan prior to the experimental task.

## Discussion

In the present study, we experimentally manipulated various aspects of the sense of body ownership across a set of conditions. In a VHI procedure within IVR, we varied the type of presented virtual object (OH, FH, Obj), its location (Congr with the location of participant’s real hand or Incongr, i.e., shifted 20 cm towards the participant’s body midline) and type of visuotactile stimulation (Syn or Asyn). In each condition, we measured the proprioceptive drift and subjective experience of ownership and disownership via a questionnaire, and compared the obtained measures between the conditions.

Firstly, in the ownership questionnaire, the mean ownership ratings were significantly higher than those in the control questions in all OH conditions (regardless of the location and type of stimulation) and in FH Syn conditions (both Congr and Incongr location), but not in FH Asyn. Therefore, in case of the subjective experience of ownership, the typical RHI pattern was replicated for the FH (i.e., the basic RHI-like condition) and the OH; for the OH, it was also present after Asyn stimulation, which is usually considered as a control condition. The Obj conditions proved to be the appropriate control, because the ownership ratings were negative in any location and stimulation type.

Furthermore, we analyzed the three ownership statements separately because each of them represented different aspects of ownership^11^. Specifically, Q1 (*“I felt as if I was looking at my own hand”*) and Q2 (*“I felt as if the Virtual Hand/Virtual Object was part of my body”*) are both considered to represent the experience of ownership, but Q1 might be more related to the component of appearance, while Q2 might focus more on the experience of embodiment per se. Finally, Q3 *(“It felt as if the touch I experienced was directly caused by the ball that was touching the virtual hand/object”*) represents the location component in the experience of ownership, i.e., the feeling that the observed stimulation of the virtual hand/object caused, or corresponded to, the tactile sensation on the real hand. The results showed that, in Q1, in Congr location, the ratings for OH were significantly higher than for FH and Obj, while in Incongr location, OH and FH were comparable and higher than Obj; moreover, the ratings were positive (representing agreement with the statement) in all OH conditions, both Syn and Asyn, and FH Syn conditions, regardless of the location. In Q2, OH and FH had comparable positive ratings in Syn conditions, which were significantly higher than for Obj; the ratings were also positive in OH Asyn conditions. In Q3, which represented location, a similar pattern was observed across all types of virtual objects in both locations, i.e., positive ratings in Syn condition and negative ratings in Asyn, with Syn being significantly higher; in Congr location, there were no significant differences between OH, FH and Obj, while in Incongr location, OH and FH were rated significantly higher than Obj. In summary, during Syn stimulation, the subjective feeling of ownership was stronger for the OH compared to the FH in the aspect of direct recognition of the seen hand (Q1), but similar to the FH in the aspect of embodiment (Q2). Interestingly, while the ratings for the FH followed the typical RHI results in form of negative ratings in the Asyn condition, it was not the case for the OH. Even though the ratings in Q1 and Q2 for the OH after Asyn stimulation were lower than after Syn stimulation, they were still positive. In turn, the location component (Q3) does not directly represent ownership, but rather the recognition of spatial and temporal congruency between the seen and the felt touches during the stimulation, which explains similar results for OH, FH and Obj.

Altogether, results of the ownership questionnaire confirm that embodiment of the FH is primarily driven by the integration of synchronous visuotactile information in case when the seen object is anatomically plausible (in accordance with^15,17,44–49^). However, the comparison between FH and OH, as well as the positive ratings in Q1 and Q2 for OH after Asyn stimulation, showed that the appearance of the hand was also relevant, to some extent. The possible role of hand appearance was previously discussed in a study by Ratcliffe & Newport^50^ that manipulated ownership of participant’s own hand and showed that multisensory integration was not sufficient for creating the feeling of ownership when the image of the own hand was distorted and misplaced. In addition, it has been shown that ownership of the own hand and the fake hand differed in terms of neural activity: for the own hand, compared to the fake hand, stronger activation of ventromedial prefrontal cortex and lateral occipitotemporal cortex, and weaker activation of the temporoparietal and ventral premotor cortices was observed^51^. Contrary to our findings, other studies did not show ownership of the own hand after asynchronous stimulation, and therefore claimed that the appearance of the hand was less relevant than the synchrony of the incoming multisensory information^34,35^. However, those studies did not compare the own hand with another hand, and the setup was not immersive (the images/videos of the participant’s hand were projected on a screen in front of her). It is also necessary to point out that, in general, we did not find differences in the sense of ownership between Congr and Incongr location of the hand/object. This might be due to the fact that, in this case, the Incongr location represented the position of the rubber hand in the typical RHI setting^9^, i.e., the hand was located in an anatomically plausible position and closer to the body midline than the real hand, and the distance between the hands was within the range that is considered suitable for inducing the illusion (e.g.,^52,53^). As in the RHI, such location allowed embodying the seen hand, and the strength of embodiment was similar to the Congr location. This further suggests that the visuotactile integration during Synch stimulation overcomes the visuoproprioceptive conflict related to the location, as long as the location is anatomically plausible. On the other hand, it could depend on the effect of seeing the virtual body from a first-person perspective (1PP), which has been demonstrated to be a sufficient condition for eliciting the embodiment illusion^24^. Indeed, previous VR studies demonstrated that, during 1PP observation, illusory ownership is felt even in case of incongruent visuo-proprioceptive information (i.e. observing a virtual arm that performs a reach-to-grasp movement while the participant’s real limb remains still^24,29,30,54^).

As for the proprioceptive drift, no drift was present in any of the Congr conditions (when the location of the virtual object corresponded with the location of the participant’s real hand) and no significant differences were observed between them. On the contrary, in Incongr conditions (when the virtual object was 20 cm closer to the body midline than the real hand), the drift was significantly higher for OH than for Obj after Syn stimulation; importantly, it was not different between Syn and Asyn stimulation within any of the Incogr conditions. The absence of proprioceptive drift in Congr conditions is to be expected, since there was no conflict between the location of the seen object and the felt real hand. In turn, in the Incongr conditions, the drift was always positive, i.e., the perceived location of the real hand shifted towards the observed virtual object. Furthermore, it did not differ between OH and FH, or between FH and Obj, despite the trend of being the highest for OH and lowest for Obj (both in Syn and Asyn conditions). In case of Incongr conditions, the absence of significant difference between Syn and Asyn stimulation of the OH and the FH contradicts the typical RHI pattern (e.g.,^9,15,45,55^). Such seemingly conflicting result for OH and FH might be explained by the difference between the RHI and the IVR setups. Indeed, as discussed in the introduction, it has been shown that, in the IVR, ownership can be evoked solely by seeing the virtual body/hand from 1PP, even without the corresponding tactile stimulation^24,25,28–30^, and the 1PP also allows maintaining the feeling of ownership after asynchronous stimulation^56^. In addition, in the IVR setup, unlike the RHI, the participants might not even be aware of the incongruence between the position of their real hand and the virtual hand that they see, again due to the stronger role of vision^32^. Therefore, vision might be given high enough weighting to overcome proprioception and tactile sensations even during asynchronous stimulation, thus evoking a similar proprioceptive drift after Syn and Asyn stimulation. As for Obj, the smallest drift and no differences between Syn and Asyn conditions were to be expected, since it was intended to be a control condition.

Furthermore, despite our predictions, the disownership questionnaire did not show any clear disownership of the participant’s real hand in any of the conditions. However, the feeling of disownership was relatively stronger in OH and FH conditions than in Obj, which is to be expected because the Obj served as a control condition. Interestingly, this pattern was comparable between Syn and Asyn stimulation for OH and FH, especially in Incongr location. This result might suggest that, similarly to the proprioceptive drift, the effect of asynchronous visuotactile stimulation could have been overcome by a strong dominance of visual information (as a consequence of the virtual setup). In general, the lack of clear disownership is not surprising, since, quite often, studies that measured subjective disownership of participant’s own hand with the same or similar questionnaire items (e.g.,^10,11^) reported barely positive, or even negative, ratings. This might be explained by the fact that disownership of the real hand is generally less vivid than ownership of the fake hand, as in the RHI/VHI the attention is focused primarily on the fake hand^57^. Alternatively, it could be partially due to the fully virtual setup of our experiment: the participants could have been always aware that the hand that they saw was virtual, even if they experienced strong ownership over it.

Finally, we found a positive correlation between the subjective level of virtual OH similarity to participant’s own real hand and the feeling of ownership of the virtual OH in Congr location after Asyn stimulation (represented by Q2). In other words, the more the participants recognized their own hand in its virtual 3D image before the experiment, the more they experienced ownership of the virtual OH in Congr location after Asyn stimulation. This further suggests that the appearance of the virtual OH, while not being the main component of the construction of body ownership, might contribute to it strongly enough to overcome, to some extent, the multisensory conflict of the Asyn stimulation. Such result contradicts the findings of Longo *et al*.^58^ that RHI was not affected by the similarity between the own hand and the rubber hand. However, the rubber hand in that study was never treated as a representation of participant’s own hand, while in our experiment, participants started the experimental tasks after acknowledging that they were seeing their “own” virtual hand.

Summarizing, all our results suggest that multisensory integration was enough to create the feeling of ownership of a virtual FH (but not a virtual Obj). In the key conditions, when the virtual hand looked like the participant’s OH, the feeling of ownership was stronger than for the FH after Syn stimulation only for the component of ownership that was more related to the appearance, and only when the virtual OH was located congruently with the real hand. Apart from that, subjective ownership of OH and FH was comparable. Crucially, unlike the FH, ownership of the OH was present even after Asyn stimulation, and it was stronger when the appearance of the virtual OH was subjectively more similar to the real hand. The results obtained for the virtual FH are in line with the neurocognitive model of body ownership^17^: FH (but not a hand-sized virtual Obj) was embodied after synchronous visuotactile stimulation, both in Congr and Incongr location, which corresponds with the previously described levels of the model, i.e., the FH was hand-shaped, placed in an anatomically plausible position and stimulated synchronously with the real hand. In case of OH, the embodiment was initially constructed through the same steps as for FH (i.e., anatomical plausibility and visuotactile synchrony), however, it is possible that an additional comparison – the one between the pre-existing body image^59,60^ and the detailed appearance of the virtual OH – took place after these steps. Such body-image-related component might not be relevant enough to create consistent differences between the embodied OH and FH even in incongruent location (at least, as measured by the proprioceptive drift and questionnaire), but it might account for the feeling of ownership of OH in the condition of visuotactile asynchrony. Importantly, it might constitute the main difference between owning one’s own body and embodying a fake body part. During the RHI, or its variations, the fake hand is embodied *despite* the mismatch between its appearance and the existing image of the real hand (e.g.,^58^). On the contrary, when it comes to one’s real body, the body image is a necessary component of bodily self-awareness^60^, and it might have a top-down effect on the multisensory integration related to the body (hence the positive ownership ratings in OH Asyn conditions).

To conclude, by directly comparing OH and FH, we showed that, while body ownership does primarily rely on the integration of temporally synchronous multisensory information, it also relies on the detailed appearance of the body (or a body part). Importantly, the IVR setup of the study, although providing the possibility to manipulate virtual OH, does not address ownership of the real body. Therefore, future studies might focus on clarifying the possible differences in body ownership of one’s own body and a virtual own body.

## Supplementary information

Supplementary information

Supplementary information2

## Data Availability

All data generated and analyzed during this study are included in this article (and its Supplementary Information).
