# Supplementary material for: Visual appearance of the virtual hand affects embodiment in the virtual hand illusion
Source: Sci Rep. 2020 Mar 25;10:5412. doi: 10.1038/s41598-020-62394-0 (PMC7096421; doi:10.1038/s41598-020-62394-0)
Supplement: Supplementary file 1 — Supplementary information [file 41598_2020_62394_MOESM1_ESM.docx]

**Visual appearance of the virtual hand affects embodiment in the virtual hand illusion**

Maria Pyasik, Gaetano Tieri, Lorenzo Pia

**Supplementary results**

*3.1 Subjective reports on ownership*

We used Wilcoxon signed rank test to analyze the questionnaire ratings (see Supplementary table 1 for medians and IQRs). Firstly, we compared averaged Ownership and Control ratings within each condition (see Figure 2A and B in the main text). The ratings in Ownership statements were significantly higher than in Control statements in all OH conditions (Congr Syn: p < .0001, *r* = .13; Congr Asyn: p = .005, *r* = .47; Incongr Syn: p < .0001, *r* = .43; Incongr Asyn: p = .004, *r* = .41), FH with Syn stimulation (Congr Syn: p < .0001, *r* = .32; Incongr Syn: p < .0001, *r* = .12) and Obj Congr Syn (p = .001, *r* = .56). In case of Obj Incongr Asyn, the ratings in Control statements were significantly higher than the ratings in Ownership statements (p = .001, *r* = .47). Finally, no significant differences were observed for FH with Asyn stimulation (both Congr and Incongr), Obj Congr Asyn and Obj Incongr Syn (.30 < p < .55).

As for the comparison of ownership ratings between Syn and Asyn stimulation conditions for each type of object, they were significantly higher after Syn stimulation compared to Asyn stimulation in every condition (OH Congr: p < .0001, *r* = .54; OH Incongr: p < .0001, *r* = .55; FH Congr: p < .0001, *r* = .48; FH Incongr: p < .0001, *r* = .57; Obj Congr: p < .0001, *r* = .59; Obj Incongr: p < .0001, *r* = .43).

The comparisons within Syn stimulation conditions showed comparable ownership of OH and FH both in Congr and Incongr location (Congr: p = .016 [N.S. after Bonferroni correction; alpha level: p = .006]; Incongr: p = .56). In turn, both OH and FH had significantly higher ownership ratings then Obj in both locations (Congr location, OH: p < .0001, *r* = .48, FH: p < .0001, *r* = .38; Incongr location, OH: p < .0001, *r* = .68; FH: p < .0001, *r* = .65). Neither OH (p = .82), nor FH (p = .16) ratings differed between Congr and Incongr location, while for Obj, the ratings in Congr location were significantly higher compared to Incongr (p = .003, *r* = .28).

Crucially, the ownership ratings were positive only for OH and FH with Syn stimulation, both Congr and Incongr location (median ± IQR ranging from 20.63±30.94 to 34.79±31.15 on a -50/+50 scale). Negative ratings in other conditions suggest the absence of subjective ownership.

In order to obtain more detailed results on ownership, we analyzed the three Ownership statements separately (see Figure 3). In Q1 (*“I felt as if I was looking at my own hand”*), the ratings in Syn stimulation were significantly higher than in Asyn in all conditions, except for Obj Incongr (p = .14): OH Congr (p = .0004, *r* = .47), OH Incongr (p = .0005, *r* = .52), FH Congr (p = .002, *r* = .27), FH Incongr (p = .0007, *r* = .38), Obj Congr (p = .0008, *r* = .53). Further comparisons within the Syn stimulation conditions showed that the ratings were the highest for OH in Congr location (OH vs FH: p < .0001, *r* = .58; OH vs Obj: p < .0001, *r* = .16), but comparable between OH and FH in Incogr location (p = .03 [N.S. after Bonferroni correction; alpha level: p = .006]. Additionally, in Incongr location, the ratings for OH were significantly higher than for Obj (p < .0001, *r* = .04). The ratings for FH were also significantly higher compared to Obj (Congr: p < .0001, *r* = .38; Incongr: p < .0001, *r* = .14). As for the comparisons between Congr and Incongr locations within each type of virtual object, there were no significant differences either for OH (p = .66), or for FH (p = .22), but the ratings were significantly higher for Obj Congr compared to Incongr (p = .0003, *r* = .59). Importantly, the ratings were positive for OH and FH in Syn conditions in both locations (medians ± IQRs ranging from 13.70±40.00 to 33.75±33.75 with the highest rating for OH Congr), and for OH after Asyn stimulation (Congr: 13.13±59.01; Incongr: 15.50±40.60), but negative for FH Asyn and all Obj conditions (medians ± IQRs ranging from -48.13±22.50 for Obj Incongr to -1.85±53.65 for FH Incongr).

In Q2 (*“I felt as if the Virtual Hand/Virtual Object was part of my body”*), similarly to Q1, the ratings were significantly higher in Syn stimulation than in Asyn in all conditions, except for Obj Incongr (p = .07): OH Congr (p < .0001, *r* = .62), OH Incongr (p = .0003, *r* = .64), FH Congr (p < .0001, *r* = .53), FH Incongr (p < .0001, *r* = .49), Obj Congr (p = .02, *r* = .40). Within the Syn stimulation conditions, OH and FH did not differ in either of the locations (Congr: p = .99; Incongr: p = .79), and both OH and FH had significantly higher ratings than Obj (Congr: OH vs Obj – p = .0001, *r* = .18; FH vs Obj – p < .0001; *r* = .44; Incongr: OH vs Obj - p < .0001, *r* = .27; FH vs Obj – p < .0001; *r* = .16). Within each type of virtual object, there were no significant differences between Congr and Incongr conditions for any of the objects (.02 [N.S. after Bonferroni correction; alpha level: p = .006] < p < .75). As in Q1, the ratings were positive for OH and FH Syn in both Congr and Incongr location (ranging from 19.10±31.90 to 26.10±41.25 with the highest rating for OH Incongr), and for OH Asyn (Congr: 7.5±45.00; Incongr: 10.30±57.80), but not for any of Obj conditions (ranging from -35.00±32.80 to -13.1±54.40).

In Q3 (“*It felt as if the touch I experienced was directly caused by the ball that was touching the virtual hand/object*”), the ratings after Syn stimulation were significantly higher than after Asyn stimulation in all conditions: OH Congr (p < .0001, *r* = .02), OH Incongr (p < .0001, *r* = .18), FH Congr (p < .0001, *r* = .28), FH Incongr (p < .0001, *r* = .34), Obj Congr (p < .0001, *r* = .09), Obj Incongr (p < .0001, *r* = .16). In Syn Congr conditions, the ratings for the three types of virtual object were not different (.01 [N.S. after Bonferroni correction; alpha level: p = .006] < p < .73), and in Syn Incongr conditions, OH and FH did not differ between each other (p = .82) but had significantly higher ratings than Obj (OH vs Obj: p < .0001, *r* = .69; FH vs Obj: p < .0001, *r* = .78). Within each type of virtual object, Congr condition did not differ significantly from Incongr condition (.02 [N.S. after Bonferroni correction; alpha level: p = .006] < p < .51). The ratings were positive in all Syn conditions (ranging from 17.35±36.85 for Obj Syn Incgr to 43.44±33.13 for OH Syn Congr) and negative in all Asyn conditions (from -44.85±20.30 for FH Asyn Congr to -38.40±24.70 for OH Asyn Congr).

*3.2. Proprioceptive drift*

According to the Wilcoxon signed rank test, proprioceptive drift was not significantly different between Syn and Asyn stimulation in any of the conditions (.41 < p < .95); see Figure 4 and Supplementary table 1. Furthermore, in the conditions with Congr location, the drift did not differ significantly between OH, FH and Obj neither in Syn (.12 < p < .67), nor in Asyn (.15 < p < .80) stimulation. As for the Incongr location, in Syn stimulation, the drift was significantly higher for OH (p = .001, *r* = .20) compared to Obj (.92±2.57). In turn, OH and FH were not significantly different (p = .23), as well as FH and Obj (p = .027, N.S. after Bonferroni correction, alpha level: p = .017]. In Incongr Asyn conditions, there were no significant differences between OH, FH and Obj (.02 [N.S. after Bonferroni correction, alpha level: p = .017] < p < .22]. To summarize, proprioceptive drift did not differ significantly between Syn and Asyn stimulation in any of the conditions; it was significantly higher for OH than for Obj in Incongr Syn condition, while no significant differences were present for Congr conditions.

It is also necessary to note that in all Congr conditions, the proprioceptive drift was negative (ranging from -1.97±4.13 cm for Obj Asyn Congr to -1.50±2.61 cm for FH Syn Congr), i.e., the perceived position of participant’s own hand shifted away from the body midline. On the contrary, in all Incongr conditions, it was positive (from .92±4.38 cm for Obj Syn Incongr to 3.18±6.77 cm for OH Syn Incongr), which suggests the perceived shift of participant’s hand towards the incongruently located virtual object and the body midline.

*3.3. Subjective reports on disownership*

For disownership questions (averaged Q7-Q9), Wilcoxon signed rank test showed no significant differences between Syn and Asyn stimulation either for OH (Congr: p = .32; Incongr: p = .34), or for FH (Congr: p = .07; Incongr: p = .79). However, for the Obj, the disownership ratings were significantly higher after Syn stimulation compared to Asyn in both locations (Congr: p = .006, *r* = .18; Incongr: p = .004, *r* = .20). In Syn Congr conditions, the ratings for OH did not differ significantly either from FH (p = .70), or from Obj (p = .02 [N.S. after Bonferroni correction; alpha level: p = .006]); however, they were significantly higher for FH compared to Obj (p = .005, *r* = .23). In Syn Incongr conditions, the ratings were comparable between OH and FH (p = .11) but were significantly higher in both those conditions compared to Obj (OH: (p < .001, *r* = .39; FH: (p < .001, *r* = .37). Finally, no significant differences were observed between Congr and Incongr location for any type of virtual object (.12 < p < .77).

Importantly, the ratings were negative in all conditions (ranging from -35.73±30.42 for Obj Incongr Asyn to -6.04±22.66 for OH Congr Asyn); see Figure 5 and Supplementary table 1. However, they were relatively higher for OH and FH conditions, especially in Incongr location, regardless of the stimulation type.

*3.4. Correlations*

We calculated Spearman rank order correlations between the subjective ratings of virtual OH similarity to participant’s own real hand and the proprioceptive drift and ownership ratings, represented both by the averaged ownership ratings (Q1-Q3) and by the three statements separately. The only significant positive correlation was observed between the subjective OH similarity and the ratings in ownership Q2 in OH Congr Asyn condition (ρ = .45, p = .002). No significant correlations were present in other conditions either for the questionnaire, or for the drift (.007 [N.S. after Bonferroni correction; alpha level: p = .004] < p < .97). Therefore, stronger embodiment of the virtual OH after Asyn stimulation was observed in the participants that were more ready to recognize their own hand in its 3D scan prior to the experimental task.

**Supplementary table 1.** Medians and IQRs of proprioceptive drift (cm), ownership and disownership ratings (-50/+50)

| **Congruent Location** | | | | | | | | | |
| --- | --- | --- | --- | --- | --- | --- | --- | --- | --- |
|  | | **OH** | | | **FH** | | | **Obj** | |
|  | Syn (median±IQR) | | Asyn (median±IQR) | Syn (median±IQR) | | Asyn (median±IQR) | Syn (median±IQR) | | Asyn (median±IQR) |
| **Proprioceptive drift** | -1.54±1.66 | | -1.56±2.90 | -1.50±2.61 | | -1.71±2.99 | -1.76±3.11 | | -1.97±4.13 |
| **Ownership (Q1-Q3)** | 34.79±31.15 | | -.42±31.25 | 20.63±30.94 | | -14.84±34.84 | -1.46±33.75 | | -25.73±38.85 |
| **Ownership Q1** | 33.75±33.75 | | 13.13±59.06 | 13.70±40.00 | | -7.20±44.70 | -24.10±51.90 | | -40.31±26.90 |
| **Ownership Q2** | 19.37±35.63 | | 7.50±45.00 | 19.10±31.90 | | -5.90±45.60 | -13.10±57.80 | | -29.10±54.40 |
| **Ownership Q3** | 43.44±33.13 | | -38.40±24.70 | 34.40±29.10 | | -44.85±20.30 | 33.80±26.00 | | -39.70±23.40 |
| **Disownership (Q7-Q9)** | -12.08±22.71 | | -13.13±25.10 | -8.75±19.17 | | -16.88±31.67 | -16.46±24.27 | | -25.42±31.15 |
| **Control (Q4-Q6)** | -18.54±28.44 | | -16.77±41.35 | -6.98±35.10 | | -17.03±31.68 | -10.00±36.88 | | -28.44±44.06 |
|  | | | | | | | | | |
| **Incongruent Location** | | | | | | | | | |
|  | | **OH** | | | **FH** | | | **Obj** | |
|  | Syn (median±IQR) | | Asyn (median±IQR) | Syn (median±IQR) | | Asyn (median±IQR) | Syn (median±IQR) | | Asyn (median±IQR) |
| **Proprioceptive drift** | 3.18±6.77 | | 2.62±5.90 | 2.30±4.36 | | 1.85±4.67 | .92±4.38 | | 1.01±3.23 |
| **Ownership (Q1-Q3)** | 27.86±30.16 | | -1.35±29.06 | 26.67±35.16 | | -9.74±34.48 | -16.09±31.41 | | -30.94±35.94 |
| **Ownership Q1** | 32.80±27.20 | | 15.50±40.60 | 18.15±37.60 | | -1.85±53.65 | -41.25±34.40 | | -48.13±22.50 |
| **Ownership Q2** | 26.10±41.25 | | 10.30±57.80 | 23.30±34.95 | | .30±54.70 | -25.80±41.14 | | -35.00±32.80 |
| **Ownership Q3** | 41.10±25.30 | | -42.80±28.40 | 40.60±20.60 | | -40.31±25.60 | 17.35±36.85 | | -38.40±24.70 |
| **Disownership (Q7-Q9)** | -6.04±22.67 | | -11.46±21.72 | -8.85±24.06 | | -7.97±34.48 | -24.69±25.83 | | -35.73±30.42 |
| **Control (Q4-Q6)** | -13.18±38.59 | | -18.70±32.71 | -9.43±44.79 | | -17.40±26.41 | -15.89±36.88 | | -21.61±45.68 |
